# Supplementary material for: Three-Way DNA Junction as an End Label for DNA in Atomic Force Microscopy Studies
Source: Int J Mol Sci. 2022 Sep 27;23(19):11404. doi: 10.3390/ijms231911404 (PMC9569629; doi:10.3390/ijms231911404)
Supplement: Supplementary file 1 [file ijms-23-11404-s001.zip › ijms-1911936-supplementary-final.pdf]

Three-Way DNA Junction as an End Label for DNA in Atomic Force Microscopy Studies

Tommy Stormberg[1], Zhiqiang Sun[1], Shaun Filliaux[1], and Yuri L. Lyubchenko[1,]

Supplememtary figures and movies

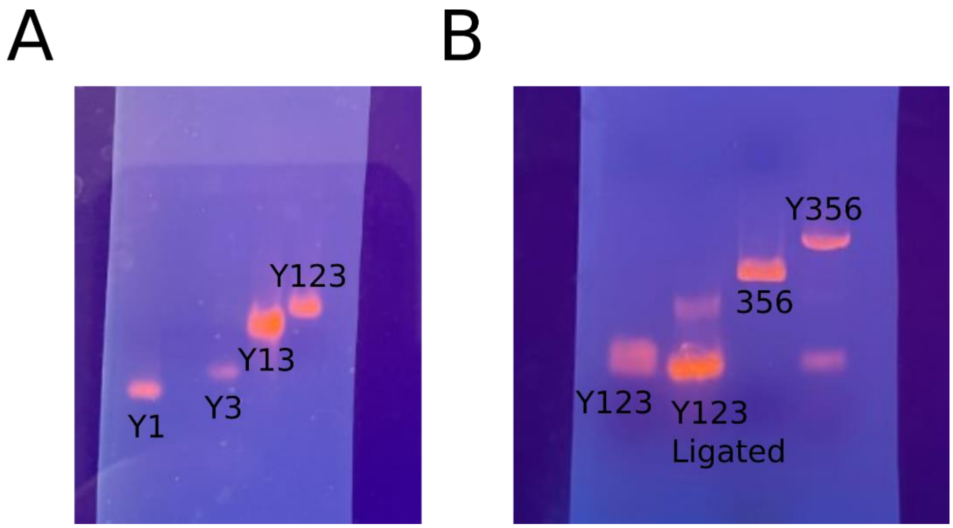

Figure S1. Agarose gels of labeled DNA complex assembly. **(A)** Gel of label assembly. The Y1 and Y3 indicated the position of single strand oligoes (The length of Y2 is similar to Y3 but not shown in gel.) The lane Y13 and Y123 are the annealing product of Y1-Y3 and Y1-Y2-Y3. The position of Y13 is higher than Y1 and Y3 but lower than the position Y123 suggested that we anneal the Y1-Y2-Y3 together. **(B)** Gel of label-DNA complex assembly. Y123 and 356 are the two parts of segment we need to ligate together. Y123 ligated shows control results for self-ligation of Y123. The main band of Y123 Ligated is same with Y123 before ligation. The Y356 is the final ligation product in which the main band is higher than both 356 and Y123.

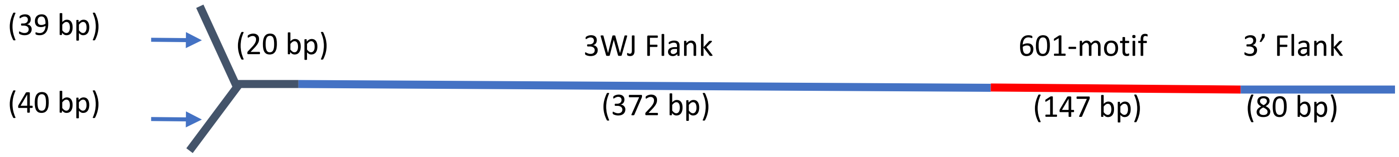

Figure S2. DNA diagram of the DNA construct in Figure 7 with a single 601-motif site (red line), 80 bp from the non-3WJ end. The 20 bp in gray, is part of the 3WJ assembly. The blue 372 bp sequence is a flank to provide the capacity for nucleosomes to bind more freely.

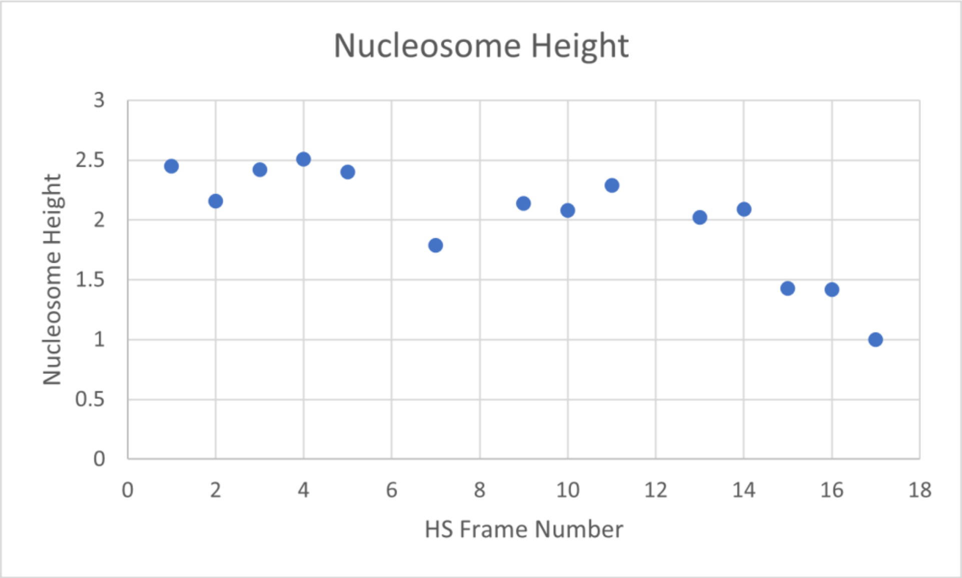

Figure S3. Nucleosome height measurement. The height values remained relatively stable for the first five frames, and then the nucleosome began to unravel, lowering the nucleosome height in the following frames.

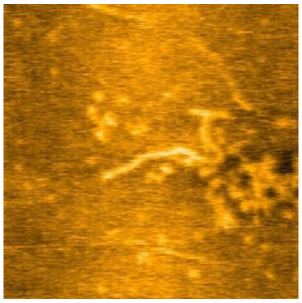

Movie S1. Dynamics of 3WJ on DNA, visualized with time-lapse high-speed AFM.
========================

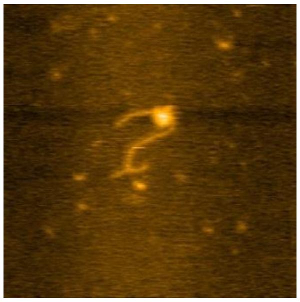

Movie S2. SDynamics of a mononucleosome on the 356 bp construct, with a visible 3WJ, visualized with time-lapse high-speed AFM.

=======================

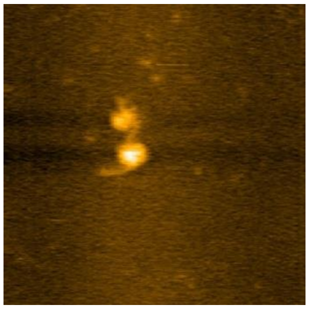

Movie S3. Dinucleosome dynamics studied on the 356 bp construct, visualized with time-lapse high-speed AFM.
